# Supplementary material for: Neighborhood level chronic respiratory disease prevalence estimation using search query data
Source: PLoS One. 2021 Jun 9;16(6):e0252383. doi: 10.1371/journal.pone.0252383 (PMC8189491; doi:10.1371/journal.pone.0252383)
Supplement: Attachment — Submitted filename: response_to reviews.pdf [file pone.0252383.s001.pdf]

Scott Counts
Senior Principal Research Manager
Microsoft Research
Redmond, WA 98052
counts@microsoft.com

April 28, 2021

PLOS One
Dr. Emily Chenette, Deputy Editor-in-Chief

Dear Dr. Chenette:

On behalf of all authors, we would like to thank the reviewer for taking the time to review our study. Specifically, we would like to thank the reviewer and editor for acknowledging the technical soundness and statistical rigor of the study and stating that only minor revisions would be needed to the current draft. We believe that our work represents very timely and significant research that will have broad impact in health sciences and epidemiology globally.

Below are the concerns raised by reviewer (in italics) and our responses.

Reviewer #1: *I recommend changing the terms in the title to chronic respiratory disease prevalence estimation. Respiratory illness is too broad, as it would include influenza, COVID-19, and other infectious diseases that were not examined. And prevalence is the correct epidemiology term for what the authors are estimating.*

We thank the reviewer for their suggestion and have updated the title of our study to reflect correct terminologies. The new title is “Neighborhood level chronic respiratory disease prevalence estimation using search query data”.

Reviewer #1: *The authors report a correlation of 0.91 in the abstract, but in the results this only relates to a full model with the outcome of asthma. Can the authors clarify in this sentence what measure of correlation is this 0.91 and also report correlations from their other outcomes? It’s probably not necessary to report all the models, just the full models with the best correlations.*

We have added updated the abstract to reflect the performance of models for both asthma and COPD.

Reviewer #1: *I recommend removing the last sentence of the abstract. This sentence does not add anything to the article, and is only a claim of “first”. Let the science speak for itself.*

We agree with the reviewer and have removed the sentence from the abstract.

Reviewer #1: *In the introduction the authors claim COPD to be the third leading cause of death in the USA. From the CDC website the third leading cause of death in the USA is accidents, and lower and chronic lower respiratory diseases are fourth (not just COPD alone). There is also an anomaly with the pandemic where COVID-19 was the third leading cause of death in 2020 and perhaps will be as high in 2021. I recommend revising this introduction as follows: “... more than 15.7 million people suffer from Chronic Obstructive Plumonary Disease (COPD). Chronic lower respiratory diseases, including asthma and COPD, are a leading cause of death in the USA, accounting for an estimated X% of all-cause mortality each year.”.*

We thank the reviewer for pointing this out and have updated the introduction to reflect up to date information.

Following is the list of changes made in the draft.

1. Title of the study changed to “Neighborhood level chronic respiratory disease prevalence estimation using search query data”.
2. Initials added to the corresponding authors email address.
3. Last line of the abstract removed as per reviewer’s suggestion.
4. Correlation value for the final models for asthma and COPD added to the abstract.
5. Line 6 of main text updated to correctly reflect the mortality of respiratory illnesses, as per reviewer’s comments.

Following is the updated data availability statement.

For privacy reasons, search engine companies cannot, and should not, ever share raw query data with the public. However, summarized data will be made available: https://github.com/microsoft/UrbanInnovation-Datasets

Following is the updated funding statement.

Microsoft Research provided support in the form of salaries for authors SC, but did not have any additional role in the study design, data collection and analysis, decision to publish, or preparation of the manuscript.

Following is the updated conflict of interest statement.

SC is affiliated with Microsoft Research. This affiliation does not alter our adherence to PLOS ONE policies on sharing data and materials.

Sincerely,

Scott Counts, PhD
